# A Permutation Entropy Method for Sleep Disorder Screening

**DOI:** 10.3390/brainsci15070691

**Published:** 2025-06-27

**Authors:** Cristina D. Duarte, Marcos M. Meo, Francisco R. Iaconis, Alejandro Wainselboim, Gustavo Gasaneo, Claudio Delrieux

**Affiliations:** 1 Departamento de Física, Instituto de Física del Sur, Universidad Nacional del Sur-Consejo Nacional de Investigaciones Científicas y Técnicas (CONICET), Bahía Blanca 8000, Argentina; cristina.duarte@uns.edu.ar (C.D.D.); marcos.meo@uns.edu.ar (M.M.M.); francisco.iaconis@uns.edu.ar (F.R.I.); 2 Lingüística y Neurobiología Experimental del Lenguaje (LyNEL), Instituto de Ciencias Humanas, Sociales y Ambientales (INCIHUSA)-Consejo Nacional de Investigaciones Científicas y Técnicas (CCT), Mendoza 5500, Argentina; awainselboim@mendoza-conicet.gob.ar; 3 Departamento de Ingeniería Eléctrica y Computadoras, Instituto de Ciencias e Ingeniería de la Computación, Universidad Nacional del Sur-Consejo Nacional de Investigaciones Científicas y Técnicas (CONICET), Bahía Blanca 8000, Argentina; cad@uns.edu.ar

**Keywords:** fractal analysis, nonlinear dynamics, permutation entropy, statistical complexity, EEG biomarkers, sleep disorder detection

## Abstract

**Background/Objectives:** We present a novel approach for detecting generalized sleep pathologies through the fractal analysis of single-channel electroencephalographic (EEG) signals. We propose that the fractal scaling exponent of permutation entropy time series serves as a robust biomarker of pathological sleep patterns, capturing alterations in brain dynamics across multiple disorders. **Methods:** Using two public datasets (Sleep-EDF and CAP Sleep Database) comprising 200 subjects (112 healthy controls and 88 patients with various sleep pathologies), we computed the fractal scaling of the permutation entropy of these signals. **Results:** The results demonstrate significantly reduced scaling exponents in pathological sleep compared to healthy controls (mean = 1.24 vs. 1.06, p<0.001), indicating disrupted long-range temporal correlations in neural activity. The method achieved 90% classification accuracy for rapid-eye-movement (REM) sleep behavior disorder (F1-score: 0.89) and maintained 74% accuracy when aggregating all pathologies (insomnia, narcolepsy, sleep-disordered breathing, etc.). **Conclusions:** The advantages of this approach, including compatibility with single-channel EEG (enabling potential wearable applications), independence from sleep-stage annotations, and generalizability across recording montages and sampling rates, stablish a framework for non-specific sleep pathology detection. This is a computationally efficient method that could transform screening protocols and enable earlier intervention. The robustness of this biomarker could enable straightforward clinical applications for common sleep pathologies as well as diseases associated with neurodegenerative conditions.

## 1. Introduction

The sleep—wake cycle is a vital physiological process in humans, and one of the most conserved biological rhythms, despite radical changes in the living conditions of our species. This cycle consists of distinct states, typically identified by their patterns and behavioral traits, including wakefulness (W), non-rapid- eye- movement (NREM) sleep, and rapid- eye- movement (REM) sleep [1]. Wakefulness and sleep are linked to different functional states of the brain, which can be observed through electroencephalographic (EEG) signals that encompass a wide range of frequencies. Alongside these electrophysiological differences, cognitive abilities undergo significant changes throughout the cycle, and sleep alterations and pathologies, including cognitive decline [2,3], mood disorders [4,5], or post-traumatic stress disorder [6,7], may significantly hamper the quality of life of subjects [8]. Sleep disorders are also linked to neurodegenerative diseases, such as Parkinson’s, where REM sleep is affected. In this sense, the detection of sleep alterations can serve as an early biomarker for these types of pathologies [9]. Sleep disorders are widely prevalent, representing a significant public health concern that requires a structured and systematic healthcare approach [10]. In this sense, early and widespread sleep pathology detection with the least invasive acquisition means is paramount. The utilization of wrist-worn raw-data accelerometers is becoming increasingly prevalent in large-scale sleep research, and is anticipated to emerge as a standard scientific instrument in the foreseeable future. However, their current accuracy in discriminating between sleep stages remains suboptimal, thereby limiting their applicability in studies focused on sleep disorders. Consequently, EEG continues to offer a more favorable balance between intrusiveness and diagnostic precision.

Despite the high prevalence and clinical impact of sleep disorders, current diagnostic methods face three key limitations: (1) reliance on labor-intensive polysomnography (PSG) with multi-channel setups, (2) fragmentation into syndrome-specific tools (e.g., apnea detectors ignore insomnia), and (3) susceptibility to inter-scorer variability in manual sleep staging. To address these challenges, we propose a unified, automated framework based on the fractal scaling of permutation entropy (PE) in single-channel EEG. Our method resolves these gaps by: (1) requiring only one EEG channel (enabling wearable applications), (2) detecting generalized pathology (e.g., RBD, insomnia, narcolepsy) without disorder-specific tuning, and (3) bypassing sleep-stage annotation entirely through whole-night PE dynamics. This approach transforms sleep disorder screening into a scalable, low-cost process while capturing nonlinear brain dynamics overlooked by traditional spectral analysis.

Given the complexity of EEG signals, traditional neuroscience approaches were based on linear signal theory, segmenting signal spectra into frequency bands and analyzing their variations during different cognitive functions and sleep states [11]. Supervised analysis and interpretation of these signals along complete sleep—wake cycles is still the main diagnostic procedure for sleep disorders [12]. Given that human-assisted analysis is often slow, expensive, and susceptible to errors, there is a clear and growing need for automated methods, which are both necessary and highly advantageous. However, linear theory only captures specific features of EEG signals, and fails to fully address the intricate, non-linear, and non-stationary nature of brain waves [13].

In Ref. [14], a review of the state of the art in sleep disorder detection is presented. Out of the 114 studies reviewed, 85 (74.6%) were primarily focused on the detection of sleep apnea, while 10 (8.8%) addressed insomnia, and each of the remaining sleep disorders was investigated in two to four studies. All these studies were solely focused on detecting only one specific sleep disorder with respect to control groups, oblivious to all other sleep disorders or divergent conditions. The primary information sources included several signals (polysomnographies, magnetic resonance images, electrocardiographies, oculograms, electromyograms, nasal airflow, etc.), apart from or combined with EEG. Sleep apnea and insomnia were the easiest to detect using EEG signals (97.14% accuracy in [15], and 90.90% accuracy in [16], respectively). Accuracies for other conditions with EEG signals were not that good.

Simultaneous identification of different sleep-related pathologies has been tackled only recently. In Ref. [17], the authors propose the analysis of an EEG microstructure scoring technique named Cyclic Alternating Pattern (CAP). They employed ensemble learning methods over wavelet-based Hjorth and entropy features extracted from monopolar C4-A1 and bipolar F4-C4 EEG channels. The model was trained with signals of healthy subjects as well as patients suffering from six different sleep disorders, provided in the CAP sleep database [18], achieving accuracies of 83% for normal subjects, and ranging from 72% to 84% for different sleep disorders.

In contrast, the field of non-linear dynamics, combined with machine learning techniques, has developed tools and models that provide insightful perspectives in complex systems and their emergent interactions [19]. For understanding and adequately assessing sleep disorders, robust methods are essential for distinguishing sleep stages within the sleep—wake cycle, and examining the microstructure of sleep waves [20]. Sleep stages range from light sleep (N1, N2) to deep sleep (N3) and REM sleep, and an evaluation of their duration and the transitions between them is paramount for the precise detection of sleep disorders, more accurate diagnoses, and targeted therapeutic interventions [21].

In this sense, Permutation Entropy (PE) is a proven and valuable tool for analyzing EEG signals (even single-channel EEG) and understanding the complexities of brain activity during different sleep stages [22,23,24]. PE is a measure of time-series complexity that quantifies the unpredictability of a signal by analyzing the distribution of ordinal patterns (OPs) within the signal, which represent the relationships between neighboring values. In general, wakefulness and REM state are characterized by higher PE values, indicating greater complexity in brain signals, while deeper sleep states show lower complexity [24,25]. Other symbolic methods, including Lempel-Ziv complexity and permutation Lempel-Ziv complexity, are also used to characterize brain activity during sleep by converting the original time series into a symbol series and then analyzing its complexity [26].

Detrended fluctuation analysis (DFA) is also a widespread analysis method for the quantification of fractal scaling (FS) properties in non-stationary signals and time series [27], and EEG signals in particular [28,29]. In Ref. [30], the FS of different sleep stages was evaluated in the different spectral bands corresponding to specific brain waves (alpha, beta, delta, and theta), with the aim of training a machine learning classifier capable of automating the sleep staging process. In Ref. [31], the FS of EEG signals of the different sleep stages of sleep apnea subjects were analyzed and compared to the corresponding ones in control subjects, showing only very slight differences. Furthermore, Ref. [32] developed a machine learning classifier to identify sleep apnea by analyzing FS in various stages of sleep in both apnea patients and control subjects. Their approach achieved a high level of classification accuracy, demonstrating the potential of computational methods for the diagnosis of sleep disorders.

In this paper, we aim to develop an automated sleep disorder detection method, from single-channel EEG signals. Our purpose is to develop the simplest, most widely applicable method that can detect a divergent sleep condition, notwithstanding the acquisition conditions (e.g., EEG channel, sampling frequency, filtering). The underlying analysis takes advantage of the mentioned properties of sleep- stage signals in terms of entropy, assessing the overall FS exponent of the PE along the entire sleep cycle. The developed model demonstrates superior classification performance compared to existing state-of-the-art studies in combined sleep disorders. The method is characterized by its simplicity and computational efficiency, making it a viable candidate for deployment in clinical applications.

## 2. Materials and Methods

### 2.1. Datasets

We used two publicly available datasets from PhysioNet: the Sleep-EDF Database Expanded [33] and the CAP Sleep Database [18]. Both datasets include expert annotations of sleep stages for each 30-s epoch, although these annotations were not used in our analysis. Epochs in which the EEG signal remained constant over time, indicating a failure in brain activity recording, were excluded from the analysis. This artifact affected 11 subjects and led to the removal of 63 epochs in total, representing approximately 0.03% of the entire dataset.

The Sleep-EDF Database Expanded contains whole-night polysomnographic recordings from two studies: the Sleep Cassette Study and the Sleep Telemetry Study. This These recordings include electrooculography (EOG), chin electromyography (EMG), and EEG recordings from Fpz-Cz and Pz-Oz, sampled at 100 Hz. The Sleep Cassette Study examined age-related effects on sleep in healthy subjects (ages 25–101) without sleep-related medication. Each participant underwent two consecutive night recordings. For this study, we analyzed all available subjects from the first night of sleep, totaling 77 participants. The Sleep Telemetry Study investigated the effects of temazepam in 22 healthy subjects with mild sleep-onset difficulties. For this study, we used the Fpz-Cz EEG channel from one night for each subject who did not receive medication, since frontal electrodes offer important advantages for distinguishing REM from Non-REM sleep, such as less muscle artifact than occipital or central leads, which is helpful in non-ideal settings [34].

The CAP Sleep Database includes 108 polysomnographic recordings, including 16 from healthy subjects and 92 from individuals with sleep disorders. The subjects with sleep pathologies include 40 diagnosed with nocturnal frontal lobe epilepsy (NFLE), 22 with REM sleep behavior disorder (RBD), 10 with periodic leg movements (PLM), 9 with insomnia (INS), 5 with narcolepsy (NARCO), 4 with sleep-disordered breathing (SDB), and 2 with bruxism (BRUX). For this study, we selected 13 healthy controls and 88 subjects with a sleep pathology, excluding bruxism due to the small number of affected subjects. EEG recordings for subjects with sleep pathologies were sampled at either 512 Hz or 256 Hz, with a 30 Hz low-pass filter, except for some NFLE subjects, whose recordings were filtered with cutoff frequencies of 128 Hz and 256 Hz. In contrast, for healthy controls, the sampling rates varied between 512, 200, and 100 Hz. Due to differences in the available channels across subjects, we selected the channels present in the majority of cases. Specifically, C4-A1 and F4-C4 were used whenever available. C4-A1 is the standard recommended by the American Academy of Sleep Medicine (AASM) for detecting differences in slow-wave activity (delta) during deep Non-REM sleep (N3) versus the low-amplitude mixed-frequency activity seen in REM [34]. In addition, we also used (whenever possible), F4-C4, for the above given reasons, i.e., the advantages of frontal electrodes for distinguishing REM from Non-REM sleep, due to less muscle artifact than occipital or central leads [34]. Table 1 summarizes the respective databases and channels chosen for the study in each condition.

### 2.2. Detrended Fluctuation Analysis

Detrended fluctuation analysis (DFA) was introduced by Peng et al. [35] to analyze the long-range temporal correlations of signals. The method can be described as follows. For $X=\{x_{k}\}$ a time series of length *N* with $x_{k}=0$ almost nowhere, the profile is defined as $Y\left(n\right)=\sum_{k=1}^{n}\left(x_{k}-\bar{X}\right)$ where $\bar{X}$ is the mean of *X*, and n=1,2,…N. The *profile* Y(n) is then divided into $N_{s}=int\left(N/s\right)$ non-overlapping segments ν of equal length *s* beginning from the first element $x_{1}$ and proceeding forward. A similar segmentation procedure is applied in reverse, starting from the last element and proceeding backwards. Consequently, a total of $2N_{s}$ segments are obtained. For each segment ν, the local trend is estimated by performing a least-squares fit to the elements within the segment, and the corresponding variance $F^{2}\left(s,\nu \right)$ is computed. The fitting procedure may involve polynomials of various orders, including linear, quadratic, or cubic functions, among others. In this study, a linear fit was employed. The fluctuation function, F(s), is obtained averaging over all segments:

(1) $$F\left(s\right)={\left\{\frac{1}{2N_{s}}\sum_{\nu =1}^{2N_{s}}\left[F^{2}\left(s,\nu \right)\right]\right\}}^{1/2}$$

This procedure is iterated for multiple values of the scale parameter *s* to examine the dependence of F(s) on *s*. The scaling behavior of the fluctuation function is assessed by analyzing the log-log plot of F(s) against *s* using standard fractal methods. If the series *X* is long-range power-law correlated, F(s) increases as a power-law,

(2) $$F\left(s\right)∼s^{\alpha }.$$

In this case, the fractal scaling exponent α can be interpreted as a long-range correlation exponent, namely an auto-affinity parameter [29].

### 2.3. Permutation Entropy

Permutation entropy (PE) is widely used in nonlinear time- series analysis due to its simplicity and effectiveness in capturing dynamical complexity [19]. This method quantifies the irregularity or unpredictability of a time series by analyzing the order relations (ordinal patterns) between consecutive values, thus making it fast to compute, less sensitive to noise and outliers, does not require assumptions about the underlying system, and works well with normalized data.

To compute PE, a time series is transformed into a sequence of ordinal patterns that represent the relative ranking of values within small overlapping segments. The probability distribution function obtained from these patterns is then used to compute the Shannon entropy. This transformation is governed by two parameters: the embedding dimension, *D*, which defines segment length, and the time lag, τ, which determines the spacing between values. Given a discrete time series $\chi =\{x_{t}\}$ of length *T*, overlapping segments of size *D* are extracted, each separated by τ, resulting in T−(D−1) segments. Each segment is assigned an ordinal pattern (OP) π based on the relative order of its *D* elements. Since there are D! possible permutations, each segment corresponds to one of these patterns. The relative frequency of the different OPs in the time series defines a probability distribution. PE is computed using the normalized Shannon entropy, which quantifies the uncertainty in the OP distribution. To ensure comparability across different embedding dimensions, the entropy is normalized by its maximum possible value, log(D!):

(3) $$\mathrm{PE}=-\frac{1}{\log D!}\sum_{i}^{D!}p\left(\pi_{i}\right)log\left(p\left(\pi_{i}\right)\right).$$

A low PE value indicates a structured, predictable process, while a high value suggests randomness or chaotic behavior. The entropy reaches its minimum value of 0 when a single OP dominates, reflecting complete determinism, and its maximum value of 1 when all OPs appear with equal probability, indicating a fully unpredictable system.

### 2.4. Scaling Exponent from PE Time Series

The time series to which we applied DFA was the PE value across epochs throughout the night, derived from the EEG signal. For each subject and channel location, the EEG signal was segmented into 30-s epochs, as this duration aligns with the standard classification of sleep stages. This segmentation resulted in an average of 1158 epochs per subject (SD = 286). PE was then calculated for each epoch based on Equation (3), with parameters τ=1 and D=4. No preprocessing filters were applied to the EEG signal prior to the computation of PE. The embedding dimension and time delay were selected based on their adequate performance in characterizing sleep stages, as reported in the literature [26]. With D=4, a total of 24 patterns are generated, sufficient to capture meaningful temporal structures while allowing reliable statistical representation of each pattern within the available data windows. For the time delay, we chose to keep τ=1 to ensure that local temporal relationships in the signal were preserved. Moreover, since our study included datasets with different sampling frequencies, using a fixed and minimal time delay allowed us to evaluate the robustness of the method across different temporal resolutions.

**Figure 1:**
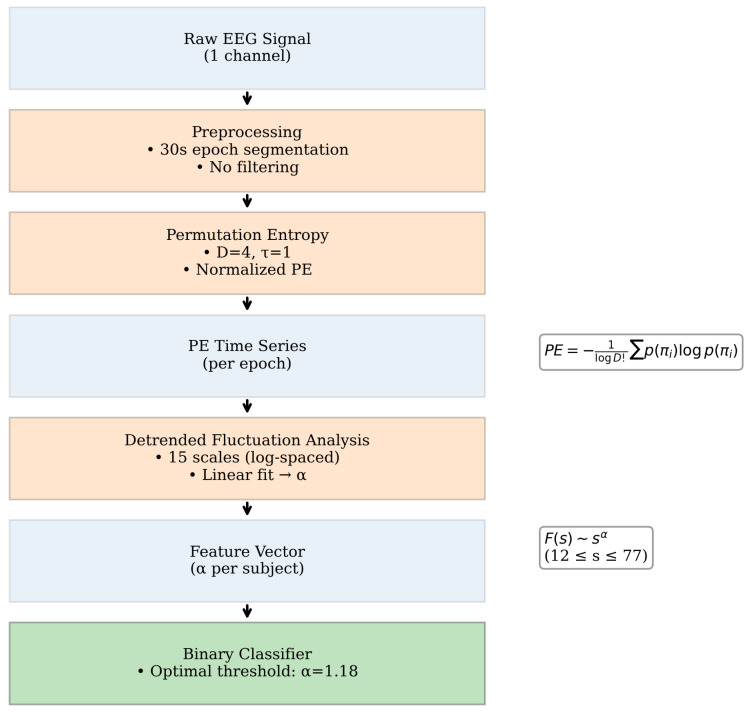
The complete processing workflow.

Once PE was obtained over time, the FS exponent α was calculated by deriving the fluctuation function F(s) from Equation (1). Fifteen segment sizes were selected in logarithmic space (base 10) within the range of 12≤s≤77 and fitted with a first-degree polynomial. The FS exponent was computed for each subject condition, and used as a feature parameter to develop a binary classifier. An optimal decision threshold was determined to maximize discriminative performance between pathological (*unhealthy*) and control (*healthy*) states. In Figure 1, we show the complete processing workflow.

To evaluate the discriminative power of the FS exponent, we first conducted statistical analyses on RBD vs. healthy controls, since RBD was the condition which that appeared to distinguish itself from healthy controls in the most pronounced manner. A parametric Student’s *t*-test was applied to assess the significance of differences in FS exponent values between RBD and healthy cohorts with EEG acquisitions from the same derivations (C4-A1, F4-C4).

To account for inter-channel variability and enhance statistical robustness, a mixed linear model (MLM) was implemented, aggregating all FS exponents across conditions. This approach ensured generalized inference while controlling for within-subject dependencies. Finally, a binary classification framework was constructed to differentiate *any* pathological sleep condition from healthy controls (e.g., an unhealthy vs. healthy classifier). This classification was performed using a threshold-based decision rule applied to the FS exponent. The optimal threshold was determined by performing a parameter sweep across a range of α values and selecting the one that maximized the F1-score. This metric, defined as the harmonic mean between the true positive rate (that is, the proportion of pathological subjects correctly identified) and the positive predictive value (PPV), provides a balanced measure of performance. By maximizing the F1-score, we aimed to minimize together false positives and false negatives in the classification process. To assess the robustness of the classification task, we introduced small perturbations around the optimal threshold and compared the resulting F1-score values. To the best of our knowledge, this work presents the first proposed framework for developing a sleep pathology detector that is not restricted to specific disorders. Instead, it enables the identification of generalized pathological sleep conditions, irrespective of their underlying etiology.

### 2.5. Computational Tools and Environment

All analyses were conducted using Python 3.8.10 on a standard personal computing environment (Intel^®^ Core™ i7-7700 CPU, Intel Corporation, Santa Clara, CA, USA, 8 GB RAM). For the computation of Permutation Entropy, we used in-house code developed by our research group, based on standard data science libraries such as NumPy (v1.26.4) and Pandas (v2.2.2). The same code has been used in previous studies [24,36], originally based on the approach described in [23]. To estimate the scaling exponent, we employed the open-source MFDFA library (v0.4.3). For data visualization, we used Plotly (v5.4.13) to generate interactive and publication-ready graphs. All data processing and analyzes analyses were carried out at the Instituto de Física del Sur (IFISUR).

## 3. Results

In this section, we present the findings of our analysis, beginning with a visualization of the temporal evolution of permutation entropy (PE) and its fractal scaling exponent α. We then proceeded to a detailed statistical analysis to assess differences between RBD and healthy subjects, followed by a classification analysis that maximizes the F1-score to distinguish between both groups. Finally, we grouped all conditions under the FS exponent to differentiate between healthy and unhealthy groups.

**Figure 2:**
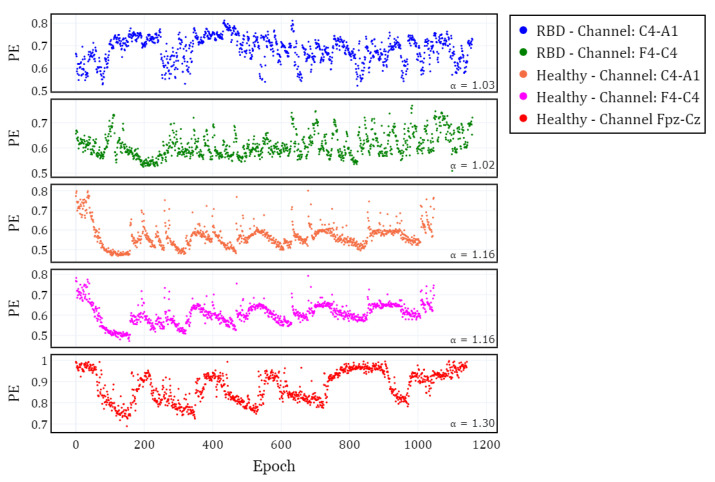
Temporal evolution of Permutation Entropy (PE) for different EEG channels in subjects with RBD and healthy controls along one night. The first two panels correspond to the C4-A1 and F4-C4 channels in one RBD subject. The last three panels correspond to the C4-A1, F4-C4 (same subject), and Fpz-Cz channels in healthy subjects.

**Figure 3:**
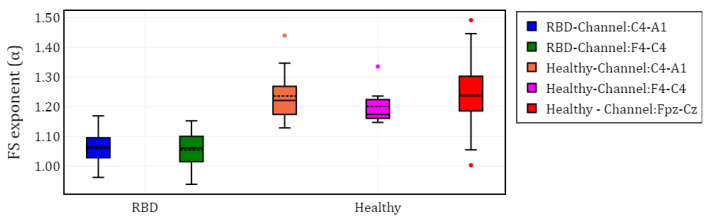
Box plot of the fractal scaling exponent (α) for different EEG channels in subjects with RBD and healthy controls.

To visualize the evolution of PE over time, we plotted the different channels (C4-A1 and F4-C4) for both a healthy subject and a subject with RBD, along with the Fpz-Cz channel for one healthy subject (Figure 2). Following the extraction of the PE time series for the selected channels in each subject, we calculated α and grouped the results by channel (C4-A1, F4-C4, and Fpz-Cz) and condition (RBD vs. healthy control). The distribution of these values is illustrated using box plots in Figure 3, providing a comparative visualization of the differences between both conditions. Additionally, the mean values of α for each condition and channel are summarized in Table 2, providing a concise overview of observed trends.

Since the CAP sleep Database includes only one night of recording and does not allow for adaptation analysis, we tested whether differences exist between first and second night data from the Sleep Cassette Study. We first assessed normality with the Shapiro-WilkShapiro–Wilk test. After confirming normality, we applied a paired *t*-test. The results showed no statistically significant difference between nights (t=1.443, p=0.153). The effect size, calculated using Cohen’s *d*, was trivial (Cohen’s *d* = 0.165). Based on this result, we used the first night of the Sleep Cassette Study in for the rest of the analysis. This ensured consistency when comparing withcompared to recordings from subjects with sleep disorders.

### 3.1. Statistical Analysis for RBD and Healthy Conditions

This subsection presents a comprehensive statistical analysis to evaluate the significance of differences in the FS exponent between RBD and healthy subjects. We first examined differences by channel within the same database using *t*-tests, followed by the application of Mixed Linear Model (MLM).

To determine whether EEG channels could be aggregated for further analysis, we first compared the α exponent across channels. First, we determined whether the EEG channels in healthy subjects (F4-C4/C4-A1 and Fpz-Cz) could be combined. To ensure the validity of our comparisons, we performed the corresponding *t*-tests after evaluating normality (Shapiro-WilkShapiro–Wilk test) and homoscedasticity (Levene test). A paired *t*-test was performed between F4-C4 and C4-A1 channels, recorded from the same subjects in the CAP database, revealing no significant difference (t=−1.998, p=0.093). We then compared these recordings with those from the Fpz-Cz channel (Sleep-EDF database), using an independent samples *t*-test, which also showed no significant differences (t=−0.492, p=0.623). Cohen’s *d* was calculated to assess size effects (Cohen’s d=0.123), indicating a small effect. Additionally, we assessed channel-related differences within the RBD group using a paired *t*-test between F4-C4 and C4-A1. No significant difference was observed (t=−1.051, p=0.305), and the effect size was very small (Cohen’s d=−0.081). These results, are consistent with those from the control group.

These results support the aggregation of EEG channels across conditions and datasets, and justify proceeding with group comparisons using the combined data.

We then examined the differences between groups within the same database. Significant differences were found between channels: t=6.038, p<0.001 for F4-C4, and t=7.155, p<0.001 for C4-A1. The effect size was large in both cases (Cohen’s d=2.62 for F4-C4, and d=2.57 for C4-A1), and the statistical power was approaching 1.0 (0.999 and 1.000), indicating a highly robust comparison. These results indicate that the scaling exponent differs between groups in both EEG channels.

To further account for individual variability, we applied a linear mixed-effects model (MLM), where the dependent variable was the α exponent, while the fixed effect was the condition (RBD vs. healthy). A random intercept per subject was included to account for individual variability in baseline α values regardless of the EEG channel. The results indicated a significant effect of group (β=−0.181, 95% CI [−0.220, −0.142], p< 0.001), suggesting that RBD subjects exhibit lower α values compared to healthy subjects. The random effect of subject showed a variance of 0.007 (SD = 0.155), indicating a relatively low variability among individuals beyond the group-level differences. These findings confirm that the difference in FS exponent between RBD and healthy subjects is statistically robust and consistent across EEG channels.

### 3.2. Subject Classification

**Figure 4:**
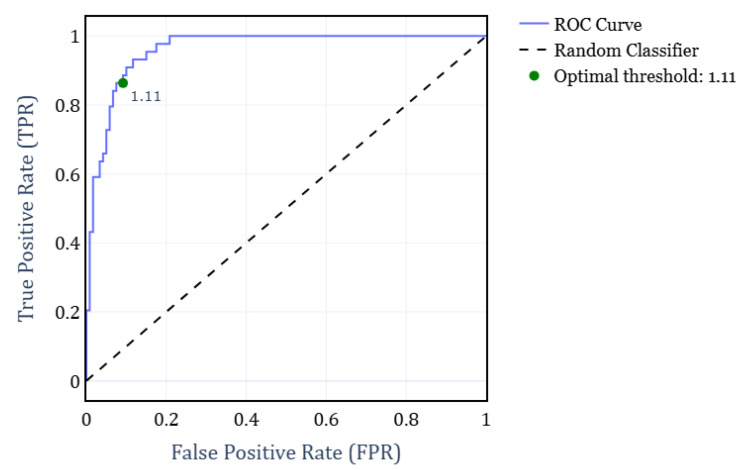
ROC curve for the classification of RBD and Healthy subjects, obtained by varying the FS exponent and grouping all subjects independently of the EEG channel.

To address the primary objective of distinguishing between RBD and healthy subjects, we developed a binary classifier that determined the subject condition solely based on their FS exponent. For this classifier, we computed the Receiver Operating Characteristic (ROC) curve under different FS thresholds. The analysis was performed by grouping all subjects according to their condition, independently of the EEG channels. Figure 4 shows the resulting ROC curve, where the optimal threshold value of α=1.11 is highlighted. This threshold was determined by maximizing F1-score, yielding a True Positive Rate (TPR) of 0.86 and a False Positive Rate (FPR) of 0.92. Consequently, it results in a weighted accuracy of 0.90, where weighted accuracy is calculated as the average of TPR and FPR, weighted by the proportion of each class.

Finally, the FS exponent was used to classify each condition (SDB, RBD, PLM, INS, NARCO) against healthy subjects. For those conditions, optimal thresholds were determined by maximizing the F1-score, using all available FS values from the EEG channels for each subject. In the case of healthy subjects, this included different combinations of channels depending on the subject (some had one channel, others had both). The performance comparison for each classifier is summarized in Table 3, and the robustness analysis for each classification task is provided in Appendix A.

**Figure 5:**
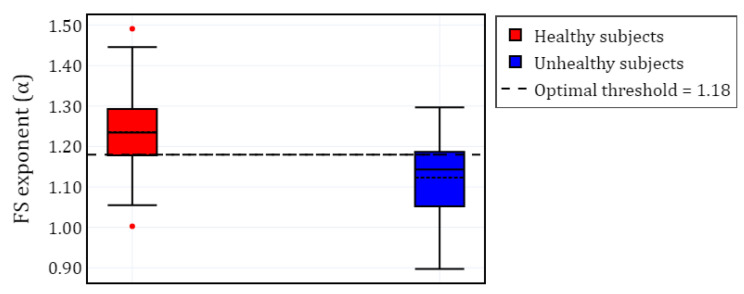
Box plot of the FS exponent (α) for healthy and unhealthy subjects (SDB, RBD, PLM, INS, and NARCO), with all α values grouped according to the available channels for each subject. The red dots represent outliers for the healthy subjects.

Furthermore, Figure 5 presents a visualization of the healthy and unhealthy populations using boxplots. The unhealthy population includes all α values from sleep pathologies, computed for the selected EEG channels. A horizontal line represents the α threshold that separates both classes.

### 3.3. Comparison with Existing Methods

To provide context for the classification performance of our method, we compared our results with those reported in recent studies reviewed by Xu et al. [14]. According to this review, most approaches focus on the detection of a single sleep disorder—typically sleep apnea or insomnia—using various physiological signals (e.g., EEG, ECG, EMG) and machine learning (ML) or deep learning (DL) classifiers.

Reported classification accuracies in those studies range from 89% to 99%, particularly for sleep apnea detection. However, these models often rely on multichannel polysomnographic data and require manual sleep- stage annotations or task-specific tuning. In comparison, our method achieves a weighted accuracy of 90% for RBD detection and a weighted accuracy of 74% for aggregated classification of multiple sleep disorders, using only single-channel EEG and no sleep- stage information. These results were obtained using a threshold-based binary classification framework applied to the scaling exponent α derived from permutation entropy.

Unlike most reviewed approaches, which are designed for single-condition detection, our method was applied to six distinct sleep pathologies using a uniform analysis pipeline. The results for each condition are summarized in Table 3. Classification performance varied across disorders, with the highest F1-score observed for RBD (0.89) and lower values for narcolepsy and insomnia (0.64 and 0.65, respectively).

## 4. Discussion

Our study demonstrates that the fractal scaling exponent of permutation entropy time series, derived from single-channel EEG, serves as a robust biomarker for distinguishing pathological sleep conditions from healthy controls. The FS exponent exhibited significant discriminative power, particularly for REM sleep behavior disorder (RBD), achieving a weighted accuracy of 90% (F1-score: 0.89) at an optimal threshold of α=1.11. Notably, this approach generalizes across multiple sleep pathologies—including insomnia, narcolepsy, and sleep-disordered breathing—with an aggregate classification accuracy of 74% (F1-score: 0.74) for all conditions combined. Given the efficiency demonstrated in REM sleep behavior analysis, this tool can serve as a robust instrument for detecting neurodegenerative diseases, such as Parkinson’s. Additionally, our method can be utilized to assess sleep quality, particularly in relation to conditions such as insomnia, and can be integrated with other traditional techniques for a more comprehensive evaluation [37].

### 4.1. Methodological and Theoretical Implications

To our knowledge, this is the first framework capable of detecting generalized sleep pathologies without requiring syndrome-specific feature engineering. Unlike prior studies focused on individual disorders (e.g., sleep apnea detection with 97.14% accuracy [15] or insomnia classification at 90.90% [16]), our method leverages the FS exponent’s sensitivity to non-linear dynamics in EEG signals. The evolution of permutation entropy across all the sleep cycles shows a noticeable higher fractal exponent in healthy subjects. This confirms other findings that show that healthier biosignals exhibit more complex fractal features as compared with unhealthier ones (see for instance the discussion in [38] regarding how EEG complexity decreases in neurological diseases, or the general idea presented by [39] that healthy systems operate at “the edge of chaos”). Also, the success of this approach aligns with growing evidence that sleep disorders share common electrophysiological signatures, such as altered long-range temporal correlations in neural activity [28].

The classification results in Table 3 demonstrate that our method is particularly effective for detecting RBD, achieving an F1-score of 0.89. The superior performance for RBD ($\alpha_{\mathrm{mean}}=1.06\pm 0.06$ vs. healthy 1.24±0.09; p<0.001) may reflect its distinct neurophysiological underpinnings, including pontine and cortical dysregulation during REM sleep [6]. In contrast, conditions such as insomnia (F1-score: 0.65) showed lower performance, possibly due to the clinical and electrophysiological heterogeneity of insomnia subtypes (e.g., sleep-onset vs. maintenance insomnia), which may not produce consistent fractal scaling patterns in a single-channel EEG. However, the FS exponent’s consistency across channels (MLM random effect variance: 0.005) underscores its applicability to diverse EEG montages, addressing a key limitation of prior channel-dependent methods.

The comparison between metrics in Table 3 shows that the weighted accuracy values are consistently higher than the F1-scores across all classification tasks. This difference occurs because weighted accuracy accounts for class imbalance (e.g., more healthy subjects than pathological cases in the dataset) by assigning proportional weights to each class. While this metric mitigates bias toward the majority class (healthy subjects), the F1-score remains the primary metric for clinical applications, as it balances precision (positive predictive value) and recall (sensitivity)—critical for avoiding false negatives in disorder screening. The high F1-score and weighted accuracy observed in the RBD vs. healthy classification task indicate robust performance despite class imbalance. Other disorders, such as SDB and narcolepsy, also achieved relatively high weighted accuracies (0.88 and 0.80, respectively), although their corresponding F1-scores (0.68 and 0.64) suggest reduced balance between correct detection and prediction reliability. These results highlight that weighted accuracy might overestimate classifier performance, whereas the F1-score provides a more conservative and informative measure. In the case of the aggregated pathology vs. healthy classification, the lower F1-score (0.74) reflects reduced generalizability when attempting to detect a wide range of disorders with distinct EEG patterns. This outcome is expected, given that the optimal α threshold differs across conditions, and applying a single threshold to heterogeneous disorders inevitably reduces classification robustness. However, the F1-score of 0.74 observed in the aggregated task remains encouraging, as it reflects reasonable performance despite the underlying diversity in EEG characteristics.

### 4.2. Clinical and Practical Advantages

This work bridges a critical gap in sleep medicine by providing a tool for *non-specific* pathology detection, which could prioritize patients for detailed diagnostics. In this sense, it presents several advantages as compared with other proposals.

- Scalability: The use of single-channel EEG and computational efficiency makes this method suitable for wearable or at-home devices.
- Generalizability: By avoiding syndrome-specific assumptions, the classifier is adaptable to comorbid or undiagnosed conditions, a critical advantage for early screening.
- Non-Invasiveness: Compared to polysomnography, this approach reduces the need for multichannel recordings, lowering costs and patient burden.
- Dataset Constraints: The method is robust with respect to heterogeneity in sampling rates and channels (Table 1). However, future validation in larger, standardized cohorts is needed (see below).
- Absence of Sleep Staging Context: While epoch-level PE was computed, the FS exponent was derived from whole-night signals, disentangling pathology effects from sleep-stage-specific dynamics (e.g., NREM vs. REM).
- Robustness to First-Night Effects: As shown in Section 3, no significant differences were found between nights in the Sleep Cassette study, indicating that our method is not affected by adaptation effects.

### 4.3. Limitations

Both the overall methodology and this specific study entail certain limitations and potential shortcomings.

- Pathology-Specific Variability: Lower accuracy for insomnia (65%) and narcolepsy (64%) suggests these conditions may require complementary information (e.g., autonomic measures).
- Limited Sample Sizes for Rare Conditions: Small cohorts (e.g., *n* = 4 for SDB, *n* = 5 for NARCO) reduce statistical power and generalizability. Aggregating pathologies mitigated this but may obscure condition-specific signatures.
- Static Thresholding: The binary classifier used a fixed FS threshold (e.g., 1.18 for all pathologies), which may ignore potential inter-individual variability in FS exponents due to age, medication, or comorbidities. This could be refined via personalized thresholds or continuous risk scoring.
- Unknown influence of gender, age, and, and comorbidities: The small sample sizes for some sleep disorder categories, limited the ability to fully analyze the variability of the fractal scaling exponent across age, gender, and comorbidities. This constrains the interpretation of the exponent as a generalized clinical biomarker.
- Comparisons with raw EEG data: While we emphasize the simplicity of applying our method directly to available EEG data, it would be necessary to compute the fractal scaling exponent using raw, unfiltered EEG signals to evaluate the potential impact of preprocessing steps such as filtering.
- Absence of external cross-validation: The fractal scaling exponent was estimated from subjects within the same database. This limits the demographic diversity and generalizability of the biomarker.

## 5. Conclusions and Further Research

Sleep is a fundamental biological process whose disruption has far-reaching consequences for cognitive health, emotional regulation, and physical well-being. The growing prevalence of sleep disorders—linked to conditions ranging from neurodegenerative diseases to cardiovascular dysfunction—demands tools that can identify pathological sleep, early, accurately, and accessibly. We presented a study that advances this goal by demonstrating that the fractal scaling properties of brain-wave complexity, quantified through permutation entropy dynamics in single-channel EEG, provide a robust and generalizable marker of sleep pathology. Our findings reveal that the loss of long-range temporal correlations in neural activity, reflected in reduced FS exponents α, is a hallmark of disordered sleep across multiple syndromes, from REM sleep behavior disorder RBD to insomnia. This insight not only bridges gaps in sleep diagnostics but also deepens our understanding of how nonlinear brain dynamics are perturbed in disease.

Furthermore, we demonstrated that our approach remains robust in the face of potential first-night effects, a known challenge in sleep research. The absence of significant differences between consecutive night recordings suggests that our method can yield reliable results from a single-night study. This practical advantage reduces diagnostic costs and enhances real-world applicability, particularly in ambulatory or wearable settings.

The quest to understand sleep—and its disorders—has long been hampered by the tension between simplicity and precision. Our study demonstrates that complexity-based metrics like the FS exponent can reconcile these goals, offering a window into the brain’s dynamic state with minimal data requirements. As sleep research increasingly embraces computational psychiatry frameworks, such methods may pave the way for a new taxonomy of sleep disorders, grounded in shared dynamical dysfunction rather than symptomatic evidence.

### 5.1. Steps Towards a Paradigm Shift

Traditional approaches to sleep diagnostics rely on polysomnography and manual staging, which are resource-intensive and often syndrome-specific. By contrast, our method leverages the inherent complexity of EEG signals—captured through PE and its FS properties—to detect pathology without requiring stage annotations, multi-channel setups, specific preprocessing, sampling rates, or filtering adjustments, etc. This aligns with a broader trend in computational neuroscience: the shift from linear, feature-engineered analyses to methods that embrace the nonlinear, non-stationary nature of brain activity. The success of the FS exponent in discriminating pathologies (e.g., RBD with 90% accuracy) underscores that sleep disorders may share underlying dynamical signatures, such as degraded signal complexity or disrupted self-organization, even when their clinical manifestations differ.

Clinically, this work addresses a critical need for simple and scalable screening tools. The FS exponent’s consistency across EEG montages and sampling rates (Table 1) suggests compatibility with emerging wearable technologies, potentially enabling at-home monitoring for high-risk populations (e.g., older adults or patients with psychiatric comorbidities). Moreover, the method’s nonspecificity—its ability to flag *any* divergent sleep condition—could help prioritize patients for detailed polysomnographies (PSG), optimizing healthcare resource allocation.

### 5.2. Theoretical Implications in Neurophysiology

The observed reduction in FS exponents α in pathological sleep, parallels findings in other neuropsychiatric conditions, where disrupted fractal scaling has been tied to degraded neural network efficiency, and alsoand with the observed fact that healthy biosignals exhibit, in general, a dense fractal nature. In our specific context, our findings may trigger speculative neurophysiological explanations. For example, the lower α values in RBD (1.06 vs. 1.24 in healthy subjects) may be a consequence of a destabilization of brainstem-cortical circuits during REM sleep, a hallmark of the disorder. Similarly, the intermediate α values in insomnia (1.17) could point to hyperarousal-driven fragmentation of sleep microstructure. These hypotheses, while speculative, highlight how the FS exponent might serve as a bridge between system-level dynamics (e.g.,, network stability) and clinical phenotypes. Future studies could test these links by correlating α with neuroimaging or molecular biomarkers.

### 5.3. Future Directions

While our results are promising, several limitations have been alreadyalready been mentioned, regarding pathology-specific variability, limited sizes, a rigid threshold setting, unclear influence of gender, age, and, comorbidities, lack of comparisons with raw EEG data, and absence of external cross-validation. In addition to addressing these issues, we are currently expanding the breadth of this research with the following aims:

1. Integration with actigraphy or heart rate variability to enhance specificity.
2. Longitudinal applications to track disease progression (e.g., RBD as a prodrome to Parkinson’s [7]).
3. Real-time implementation in clinical wearables for continuous monitoring.
4. The use of sleep-stage context, which might enhance the classifier’s specificity.
5. The fixed threshold could be refined using population-adjusted or adaptive models to account for inter-individual variability.
6. Collaborate with initiatives like the National Sleep Research Resource (NSRR) to access larger, harmonized datasets.
7. Combine FS exponents with time-domain features (e.g., spectral power, Hjorth parameters) to capture complementary information.
8. Analyze the PE time series in more complex terms, for instance, multifractality.

A focus on embedding these tools into scalable platforms would validate them in real-world settings, and explore their mechanistic underpinnings. By doing so, we move closer to a future where sleep health is monitored as routinely as blood pressure, preventing disease before it takes hold.

## Figures and Tables

**Table 1:** Summary of EEG sources, including the available EEG channels, Sampling Rates (SR), and lowpass (LP) filtering.

Condition	Database	Subject Count	EEG Channels	SR (Hz)	LP Filter (Hz)
SDB	CAP	1	C4-A1 and F4-C4	512	30
		3	C4-A1 and F4-C4	256	30
NFLE	CAP	29	C4-A1 and F4-C4	512	30
		9	C4-A1 and F4-C4	256	30
RBD	CAP	22	C4-A1 and F4-C4	512	30
PLM	CAP	9	C4-A1 and F4-C4	512	30
		1	C4-A1 and F4-C4	256	30
INS	CAP	7	C4-A1 and F4-C4	512	30
		2	C4-A1 and F4-C4	256	30
NARCO	CAP	5	C4-A1 and F4-C4	512	30
Healthy	CAP	6	C4-A1 and F4-C4	512	30
		1	C4-A1 and F4-C4	200	100
		1	C4-A1 and F4-C4	100	50
		3	C4-A1	200	100
		1	C4-A1	100	50
		1	F4-C4	200	100
	Expanded	99	Fpz-Cz	100	50

**Table 2:** Mean value ($\alpha_{\mathrm{mean}}$) and standard deviation (SD) of the scaling exponent α for each EEG channel in healthy and sleep pathology conditions.

Condition	Channel	$\alpha_{\mathrm{mean}}$	SD
SDB	C4-A1	1.12	0.02
	F4-C4	1.12	0.04
NFLE	C4-A1	1.15	0.11
	F4-C4	1.15	0.11
RBD	C4-A1	1.06	0.06
	F4-C4	1.05	0.06
PLM	C4-A1	1.11	0.06
	F4-C4	1.10	0.06
INS	C4-A1	1.17	0.01
	F4-C4	1.18	0.02
NARCO	C4-A1	1.16	0.01
	F4-C4	1.16	0.01
Healthy	C4-A1	1.24	0.09
	F4-C4	1.20	0.06
	Fpz-Cz	1.24	0.09

**Table 3:** Classifier performance for unhealthy vs. healthy subjects, by condition, and in aggregate across all pathologies. The optimal threshold for the α value is also presented.

Classification	Threshold	F1-Score	Weighted Accuracy
SDB vs. healthy	1.14	0.68	0.88
RBD vs. healthy	1.11	0.89	0.90
PLM vs. healthy	1.11	0.78	0.89
INS vs. healthy	1.17	0.65	0.77
NARCO vs. healthy	1.17	0.64	0.80
All pathologies vs. healthy	1.18	0.74	0.74

## Data Availability

The code developed for this study will be made available upon the acceptance for publication due to being part of an ongoing study.

## Appendix A. Evaluation of Classification Threshold Robustness

To assess the robustness of the classification threshold, we applied a variation of ±50% of the standard deviation of the scaling exponent α around the optimal threshold for each sleep pathology (see Table 2). For each perturbed threshold, the F1-score was recalculated. Results are shown in Table A1).

**Table A1:** Changes in F1-score under threshold perturbations (±50% of the standard deviation of the decision variable) for each classification task.

Classification	F1-Score ($\alpha_{\mathrm{opt}}$−0.5SD)	F1-Score ($\alpha_{\mathrm{opt}}$)	F1-Score ($\alpha_{\mathrm{opt}}$ + 0.5SD)
SDB vs. healthy	0.678	0.679	0.669
RBD vs. healthy	0.808	0.886	0.869
PLM vs. healthy	0.673	0.757	0.732
INS vs. healthy	0.540	0.648	0.646
NARCO vs. healthy	0.599	0.644	0.629
All pathologies vs. healthy	0.730	0.739	0.722

As expected, the fallout is more noticeable on the lower threshold value ($\alpha_{opt}$−0.5SD), given that this increases the recall of the classifier and thus several false positives arise. On the contrary, the higher value ($\alpha_{opt}$ + 0.5SD) increases the specificity of the classifier, giving rise to a smaller amount of false negatives (as compared with the former threshold). This outcome suggests that the performance of the classification method is stable under feasible perturbations of the decision threshold. Although slight variations in F1-score can be observed, particularly in the classification of PLM and INS, the overall changes are minor and do not compromise the validity of the approach. This implies that the method does not rely on a fine-tuned threshold and maintains robustness across a range of threshold values. Therefore, the proposed classification strategy can be considered reliable even when applied to data with small variations in the distribution of the decision variable.
